# Molecular Alterations in Thyroid Cancer: From Bench to Clinical Practice

**DOI:** 10.3390/genes10090709

**Published:** 2019-09-13

**Authors:** Elena Tirrò, Federica Martorana, Chiara Romano, Silvia Rita Vitale, Gianmarco Motta, Sandra Di Gregorio, Michele Massimino, Maria Stella Pennisi, Stefania Stella, Adriana Puma, Fiorenza Gianì, Marco Russo, Livia Manzella, Paolo Vigneri

**Affiliations:** 1 Department of Clinical and Experimental Medicine, University of Catania, 95123 Catania, Italy; 2 Center of Experimental Oncology and Hematology, A.O.U. Policlinico-Vittorio Emanuele, 95123 Catania, Italy; 3 Department of Medical Oncology A.O.U. Policlinico-Vittorio Emanuele, 95123 Catania, Italy; 4 Department of Clinical and Experimental Medicine, Section of Endocrinology, University of Catania, Garibaldi-Nesima Medical Center, 95122 Catania, Italy

**Keywords:** differentiated thyroid cancer, anaplastic thyroid cancer, medullary thyroid cancer, radioactive iodine resistance, molecular alterations, targeted therapy, tyrosine kinase inhibitors, mTOR inhibitors, immunotherapy, clinical trials

## Abstract

Thyroid cancer comprises different clinical and histological entities. Whereas differentiated (DTCs) malignancies are sensitive to radioiodine therapy, anaplastic (ATCs) and medullary (MTCs) tumors do not uptake radioactive iodine and display aggressive features associated with a poor prognosis. Moreover, in a majority of DTCs, disease evolution leads to the progressive loss of iodine sensitivity. Hence, iodine-refractory DTCs, along with ATCs and MTCs, require alternative treatments reflective of their different tumor biology. In the last decade, the molecular mechanisms promoting thyroid cancer development and progression have been extensively studied. This has led to a better understanding of the genomic landscape, displayed by thyroid malignancies, and to the identification of novel therapeutic targets. Indeed, several pharmacological compounds have been developed for iodine-refractory tumors, with four multi-target tyrosine kinase inhibitors already available for DTCs (sorafenib and lenvatinib) and MTCs (cabozantib and vandetanib), and a plethora of drugs currently being evaluated in clinical trials. In this review, we will describe the genomic alterations and biological processes intertwined with thyroid cancer development, also providing a thorough overview of targeted drugs already tested or under investigation for these tumors. Furthermore, given the existing preclinical evidence, we will briefly discuss the potential role of immunotherapy as an additional therapeutic strategy for the treatment of thyroid cancer.

## 1. Introduction

Thyroid cancer represents 2.5% of all cancers and about 90% of endocrine tumors [1]. The incidence of this neoplasia, three-fold higher in women and in individuals aged 25 to 65 years [2], has increased by 4.5% per year over the last decade [3], possibly because of diagnostic techniques improvement, environmental (e.g., radiation, pollution) and lifestyle changes [4,5]. Histologically, thyroid tissue consists of two different epithelial populations, the follicular and para-follicular [also known as C (clear)] cells, with different embryological origins and function. Follicular cells, organized in functional units called follicles, synthetize and secrete thyroid hormones (thyroxine and triiodothyronine), whereas C-cells, nestled between the follicles, secrete the hormone calcitonin [6].

Thyroid cancers are classified in follicular-derived and C-cell-derived, according to their cell of origin. Follicular-derived thyroid malignancies are subdivided in differentiated (DTCs) and anaplastic (ATCs) thyroid carcinomas. DTCs include the papillary (PTCs) and follicular (FTCs) histotypes, Hurtle cell thyroid cancers and the more aggressive poorly differentiated carcinomas (PDTCs) [7,8]. ATC accounts for about 2% of all thyroid cancers and is constituted by undifferentiated cells with very low similarity to normal thyroid tissue [9].

Lastly, medullary thyroid cancer (MTC) stemming from para-follicular C-cells represents 2–5% of all thyroid carcinomas [10]. It is a neuroendocrine tumor that can be either sporadic (75%) or familial (25%) the latter defined as part of Multiple Endocrine Neoplasia type 2 (MEN2) syndrome [11]. MEN2 comprises MEN2A, which features familial MTC (FMTC) [12] or MTC plus pheochromocytoma and hyper-parathyroidism/parathyroid adenoma, and MEN2B also characterized by MTC and pheochromocytoma, but with mucosal ganglioneuromas and marfanoid habitus.

The vast majority of DTCs usually display an indolent course, and standard upfront treatment involves surgery followed by adjuvant hormone replacement and radioactive iodine (^131^I-based RAI) therapy for high-risk diseases [13]. Despite their overall good prognosis, 10–20% of DTC patients present distant metastases at diagnosis or will subsequently develop them [14]. The majority of these patients are eligible for RAI, with a 40% chance of achieving a complete and durable response [15]. However, the remaining 60% display primary or acquired resistance to RAI (RAIR), thus needing further treatment options [16].

Unlike DTC, ATC grows rapidly and does not maintain the features of follicular cells, including iodine uptake. Indeed, ATC shows suppression of the sodium iodide symporter (NIS) expression/function and RAI refractoriness. Hence, radiotherapy and chemotherapy are the only treatment options for this disease, even though reported outcomes are dismal [17,18].

Since para-follicular cells intrinsically lack ^131^I avidity, thyroidectomy with subsequent hormone replacement is the treatment of choice for localized MTC. However, targeted therapies or, less frequently, chemotherapy may be an option for locally advanced or metastatic patients [19].

Thyroid cancers are characterized by molecular alterations, such as activating/inactivating mutations, rearrangements and copy number variations in genes responsible for cell proliferation, differentiation and apoptosis [20]. In recent years, the discovery of disease-specific molecular targets has led to the approval of new drugs (e.g., sorafenib, lenvatinib, vandetanib and cabozantinib), which are currently available for metastatic RAIR DTCs and MTCs [21,22]. Aim of this review is to discuss the molecular alterations associated with DTCs, ATCs and MTCs and provide an update on recently published studies and ongoing trials testing targeted therapies and immunotherapy in advanced thyroid carcinomas.

## 2. Molecular Alterations in Thyroid Cancers

**Figure 1:**
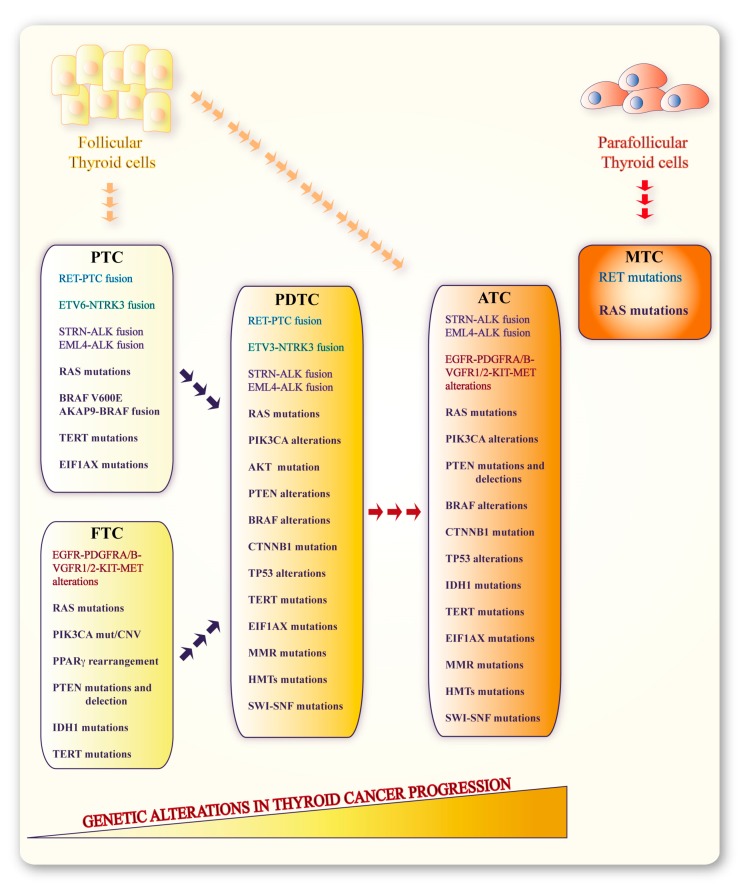
Genetic events involved in thyroid carcinogenesis. Papillary thyroid carcinomas (PTC), follicular thyroid carcinomas (FTC) and anaplastic thyroid carcinomas (ATC) originate from thyroid follicular cells and are characterized by molecular alterations (mutations, deletions, gene fusions) involving genes and proteins impinging upon different cellular pathways. The transition from PTC/FTC to poorly differentiated (PDTCs) and ATCs is attributed to additional molecular alterations. Medullary thyroid carcinoma (MTC) originates from para-follicular C-cells and is prevalently characterized by *RET* or *RAS* mutations.

As thyroid cancer progresses, the accumulation of molecular alterations disrupting multiple normal cell functions results in RAIR development, due to impaired NIS expression [23,24,25]. Indeed, dysregulation of different receptor-tyrosine kinase (RTK)-dependent signaling and proliferation pathways—such as the mitogen-activated protein kinase (MAPK), the phosphoinositide 3 kinase (PI3K), the Wingless/Integrated (WNT), the p53 and p73 pathways—are involved in the multistep tumorigenic process of thyroid cancer [25,26,27] (Figure 1). Alterations of these cascades can be linked to different mechanisms, including genetic and epigenetic modifications in pathway receptors and effectors [28,29]. Moreover, distinct, mutually exclusive molecular alterations may be associated with specific disease stages or histotypes [30].

In order to better classify the molecular alterations detected in thyroid cancer, we will initially discuss RTK-related upstream signaling pathways involved in tumorigenesis and subsequently focus on the effectors of these pathways. Finally, we will describe alterations contributing to thyroid carcinogenesis that involve pivotal cellular functions.

### 2.1. Alterations in RTKs

Rearrangements, copy number gains and point mutations are the genetic alterations more frequently observed in RTKs. The main consequence of these alterations is increased protein expression and downstream activation of different signaling pathways involved in thyroid cancer progression [31,32,33].

**Figure 2:**
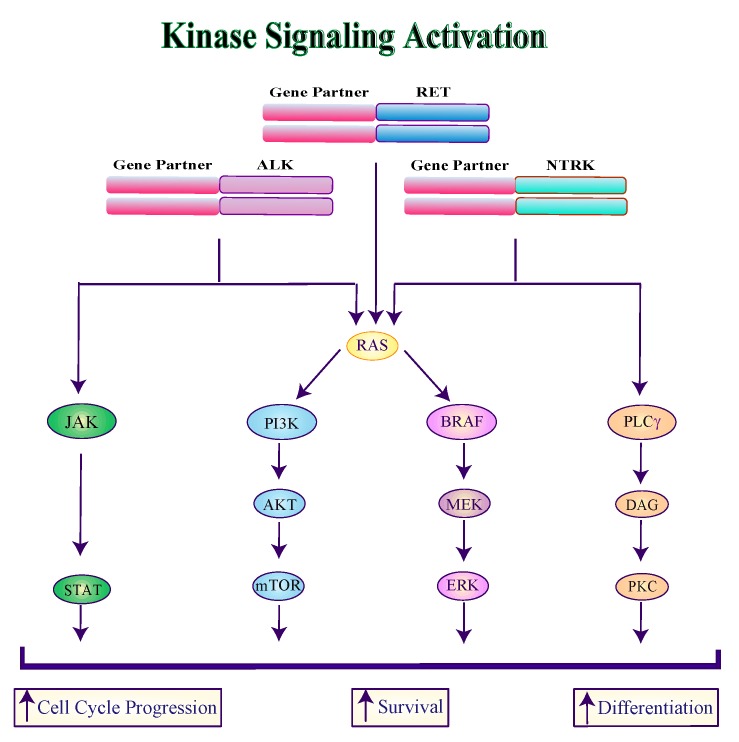
Schematic overview of ALK, NTRK and RET fusion proteins signaling. The indicated fusion proteins activate the JAK/STAT, PI3K, MAPK and PLCγ pathways involved in cell cycle progression, survival and differentiation. ALK, anaplastic lymphoma kinase; NTRK, neurotropic tropomyosin receptor kinase; RET, rearranged during transfection; RAS, Rat Sarcoma; JAK, Janus kinase; STAT, signal transducers and activators of transcription; PI3K, phosphoinositide 3-kinase; AKT, V-Akt Murine Thymoma Viral Oncogene Homolog; mTOR, Mammalian Target of Rapamycin; BRAF, B-Raf proto-oncogene; MEK, Mitogen-Activated Protein Kinase; ERK, Extracellular Signal-Regulated Kinase; PLCγ, phospholipase C-γ; DAG, Diacylglycerol; PKC, protein kinase C.

**ALK:** The *anaplastic lymphoma kinase* (*ALK*) may undergo both activating mutations in exon 23 (L1198F and G1201E) [34] and gene rearrangements (especially in PTCs, 1–3%) [35]. While the most common rearrangement involves the *striatin* (*STRN*) gene (*STRN-ALK*), *ALK* may also rearrange with the *echinoderm microtubule-associated protein-like 4* (*EML4*) gene (*EML4-ALK*) [36,37]. Furthermore, two novel ALK fusions have been recently identified (*GTF2IRD1-ALK* and *MALAT1-ALK*) [30]. Both ALK mutants and cytoplasmic ALK fusion proteins promote the activation of MAPK, PI3K and JAK/STAT downstream pathways (Figure 2). ALK mutations and rearrangements are mainly found in PDTCs and ATCs, as they contribute to disease progression and aggressiveness [34,38,39,40].

**NTRK:** The *Neurotrophic tyrosine kinase receptor* (*NTRK*) gene encodes for the tropomyosine-related kinase (Trk)-family of proteins known as TrkA (encoded by *NTRK1*), TrkB (encoded by *NTRK2*) or TrkC (encoded by *NTRK3*). In PTCs, chromosomal rearrangements, due to environmental factors (e.g., ionizing radiations) cause *NTRK* fusions with different partners [41]. Cytoplasmic Trk fusion proteins activate downstream signaling via PI3K, MAPK and phospholipase C-gamma (PLCγ) that control cell-cycle progression, proliferation, apoptosis and survival (Figure 2). The major *NTRK* fusions occur in PTCs between *NTRK3* and *ETS Variant 6* (*ETV6-NTRK3*) [41], but have also been identified in 25% of pediatric PTCs [42]. However, in PTCs, *NTRK1* may also rearrange with *tropomyosin 3* (*TPM3)*, *translocated promoter region* (*TPR)* and *trafficking from ER to Golgi regulator* (*TFG)* [43].

**Figure 3:**
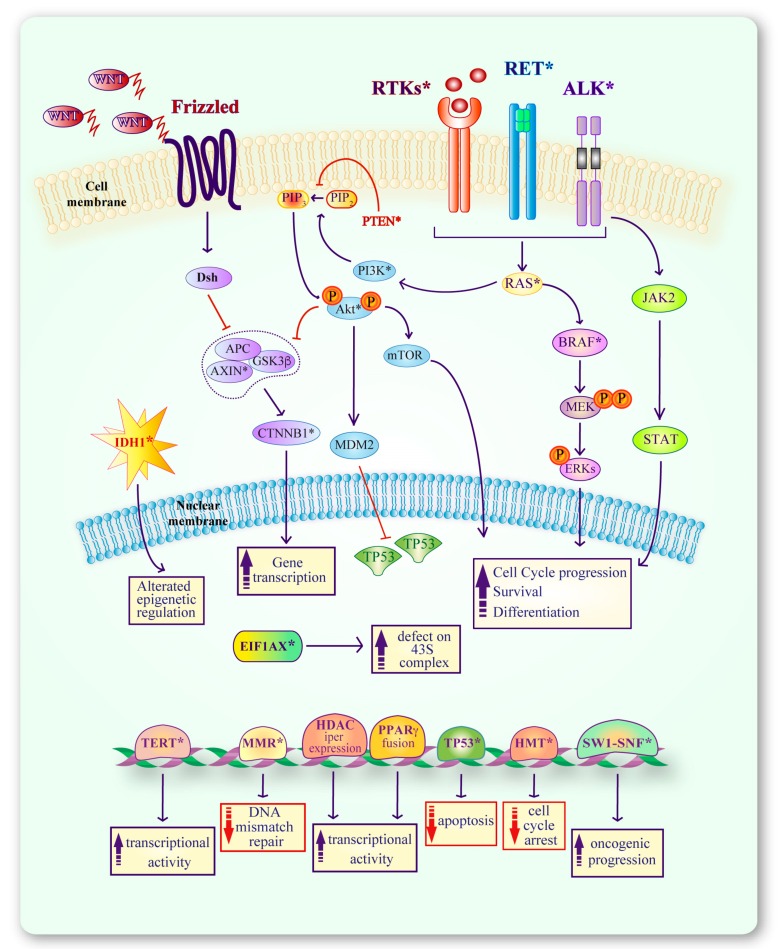
Molecules and cellular pathways that contribute to thyroid cancer development. Alterations in ALK, RET or other RTKs up-regulate RAS thereby pathologically modulating the MAPK and PI3K pathways that favor thyroid cell survival, de-differentiation and improper gene transcription. Mutations in the WNT pathway (CTNNB1, AXIN) and in other molecules (TERT, PPARγ, HMT, SW1-SNF, TP53) promote oncogenic activity, reduce apoptosis and compromise DNA repair (MMR). IDH1 or EIF1AX mutations alter epigenetic mechanisms or cause a defective assembly of the 43S-complex, respectively. Additional mutations in specific genes (BRAF, PTEN) promote the transition from differentiated to undifferentiated thyroid cancer. * indicates genes directly involved in thyroid carcinogenesis

**RET:** The *Rearranged during Transfection* (*RET*) proto-oncogene is frequently altered in thyroid cancer. Specifically, gene translocations identified as *RET/PTC* rearrangements are prevalent in PTCs (5–25%), while *RET* mutations are the primary molecular mechanism underlying MTC tumorigenesis [44]. These events share a common downstream effect as they lead to RET constitutive activation and improper stimulation of both the MAPK and PI3K pathways (Figure 3). To date, at least 19 different rearrangements between the 3’ portion of *RET* (containing the tyrosine kinase domain) and the 5’ portion of partner genes have been described, [30]. The most frequent fusions are *RET-PTC1* (60% of RET-rearranged PTCs), involving the *coiled-coil domain-containing gene 6* (*CCDC6*), *RET-PTC3* (30%), generated by the fusion with the *nuclear receptor co-activator 4* (*NCOA4*) and, less frequently (5%) *RET-PTC2*, involving the *protein kinase cAMP-dependent type I regulatory subunit alpha* (*PRKAR1A*). These rearrangements determine loss of the RET transmembrane domain leading to the cytosolic localization of the protein [45]. While RET recombinations are more frequent in young PTC patients (45–60%) as well in radiation-related tumors (up to 80%) [46], they have also been identified in PDTCs, ATCs and MTCs [35].

Several gain-of-function germline or somatic *RET* mutations arise in hereditary or sporadic MTC patients, respectively [47,48]. In most cases, mutations causing MEN2A involve cysteines within the cysteine-rich extracellular domain (exons 10 and 11) at codon 634 (C634R; 80% frequency) or codons 609, 611, 618, 620 and 630 [49]. These single nucleotide variations cause constitutive dimerization and activation of the receptor, in a ligand-independent manner. The most frequent substitution found in MEN2B patients (95%) is the M918T mutation in exon 16 that induces constitutive kinase activation in the absence of dimerization [50]. Other rare mutations involve codons 634, 691, 838, 883 and 904 [48]. In 95% of FMTC patients, mutations occur at codon 620, although rare substitutions have been reported in other codons, including 611 and 618 [49]. Finally, about 40% of sporadic MTC patients present a somatic *RET* mutation that in 80% of cases is M918T [51].

**Others RTKs:** Copy number gains in several other RTKs [*epidermal growth factor receptor **(EGFR**), platelet-derived growth factor receptor A/B (**PDGFRA/B**), vascular endothelial growth factor receptor 1,2 (**VEGFR1,2**), Mast/Stem Cell Growth Factor Receptor Kit (**c-KIT**) and MET Proto-Oncogene, Receptor Tyrosine Kinase (**MET**)*] have been identified in different subtypes of thyroid cancer [25,31,52]. These alterations are associated with increased phosphorylation of AKT, leading to activation of the PI3K pathway [31] (Figure 3). Interestingly, no mutations have been reported in these genes, with the exception of a single case of PTC displaying the G735S EGFR point mutation, which causes a conformational change of the kinase domain leading to its constitutive activation [53]. *Fibroblast growth factor receptor 2 (**FGFR2**)* and *FMS-like tyrosine kinase 3 (**FLT3**)* missense mutations have been identified in 11% and 17% of PDTCs, respectively [54]. Lastly, *FGFR2* fusions may occur in PTCs with very low frequency (<1%) [30,35], while *FGFR4* may be overexpressed in PTCs, FTCs and MTCs [52].

### 2.2. Alterations in the PI3K Pathway

Enhanced PI3K signaling is a common feature of thyroid cancer, in particular in the FTC subtype [25] (Figure 3). Alterations in this pathway involve the GTPase RAS, the alpha catalytic subunit of phosphatidylinositol-4,5-bisphosphate 3-kinase (PIK3CA), the serine-threonine protein kinase AKT and the phosphatase and tensin homolog phosphatase (PTEN). While *RAS* mutations are considered an early event in thyroid cell tumorigenesis, alterations in other downstream effectors of the pathway characterize the less differentiated thyroid cancer histotypes [55].

**AKT:** Activating mutations in *AKT* (e.g., the single hotspot E17K mutation promoting constitutive localization to the plasma membrane) inhibit apoptosis in thyroid cells [39]. *AKT* copy number gains have also been reported [31]. As for PIK3CA, *AKT* mutations represent a late event in thyroid tumorigenesis; hence, they are more frequent in PDTCs (19%) [56].

**PIK3CA:** PIK3CA may exhibit activating mutations or undergo copy number gains. Missense mutations take place in exons 9 and 20 (E542K, E545K and H1047R) and are less frequent than amplifications occurring at chromosome site 3q26.3 [57]. These events increase PIK3CA protein expression, yet their tumorigenic role is not well defined. PIK3CA mutations and copy number gains are mutually exclusive in WDTCs, but can co-occur in less differentiated tumors, where they drive disease progression [58,59]. PIK3CA alterations are common in ATCs (18%) and less frequent in FTCs (1%) and PDTCs (2%) [31,39].

**PTEN:** Alterations involving the tumor suppressor *PTEN* lead to constitutive activation of the PI3K pathway, causing an increase in cell proliferation, motility and protein synthesis. *PTEN* inactivating mechanisms include mutations, loss of heterozygosis, deletions and epigenetic modifications, resulting in the loss of PTEN expression [60]. *PTEN* alterations are described in FTCs [31], and their frequency increases with thyroid tumor progression (4% in PDTCs and 15% in ATCs) [39].

**RAS:** The RAS oncoprotein is a common effector of both the PI3K and MAPK pathways, although *RAS* mutations in thyroid cancer prevalently alter the PI3K cascade [31]. Alterations can occur in codons G12, G13 and Q61 and may involve one of the three *RAS* genes (*KRAS*, *HRAS* and *NRAS*), albeit the latter is predominantly mutated in thyroid tumors [61]. The effect of these mutations is to lock RAS in its active GTP-bound form. *RAS* point mutations mainly characterize FTCs (30–50%), but are also frequent in *RET* wild-type sporadic MTCs (10–45%), since point mutations in these two proteins are mutually exclusive [62]. Furthermore, *RAS* mutations have also been found in PTCs (5%), PDTCs and ATCs (both around 25%) [39,61].

### 2.3. Alterations in the MAPK Pathway

The MAPK pathway is frequently altered in thyroid cancer, particularly in PTCs [63]. In most cases, mutations involve *RAS* (previously described) and the *B-Raf proto-oncogene* (*BRAF*), in addition to the upstream receptors described above, which activate different signaling cascades (Figure 3).

***BRAF*:** Alterations in *BRAF* are an early tumorigenic event in PTCs (40–80%) although they have also been reported in PDTCs (5–35%) and ATCs (10–50%) [64]. Point mutations in this serine/threonine kinase activate the MAPK pathway resulting in loss of differentiation, tumor progression and inhibition of apoptosis [65,66]. The most common *BRAF* mutation is the V600E substitution, found in 45% of PTCs, which causes constitutive activation of the proto-oncogene [67]. Rare mutations may occur around codon 600, the most frequent the K601E substitution, and display an inferior oncogenic potential [30,67]. *BRAF* fusions have also been found in radiation-associated PTCs and, at a lower frequency, in PDTCs and ATCs [30,35]. The first to be identified was the *AKAP9-BRAF* rearrangement, found in 10% of radiation-induced PTCs, resulting in a fusion protein lacking the auto-inhibitory N-terminal portion of BRAF that exhibits elevated kinase activity [68].

### 2.4. Alterations in the WNT Pathway

Mutations in genes encoding members of the WNT signaling pathway—i.e., *Catenin Beta 1* (*CTNNB1*), *AXIN1* and *Adenomatous Polyposis Coli* (*APC*)—are hallmarks of less differentiated thyroid carcinomas, in particular, ATCs [69]. Mutations in the transcription factor *CTNNB1* are frequent events (>60%) [70,71], that modify its phosphorylation leading to protein stabilization because of reduced degradation [69]. These alterations become more frequent with loss of thyroid cancer differentiation (25% in PDTCs and 60–65% in ATCs) [25] (Figure 3).

### 2.5. Alterations in the TP53 Pathway

The tumor suppressor TP53 is a transcription factor involved in the control of the cell cycle and apoptosis (Figure 3). More than 75% of *TP53* mutations are small nucleotide changes that inactivate the protein’s function. These changes are mostly located in the DNA-binding domain (residues 92–292) [72]. Considering the high prevalence of *TP53* mutations in PDTCs (10–35%) and ATCs (40–80%), *TP53* inactivation is considered a final step in tumor progression. Indeed, p53 deficiency, in association with activating mutations of oncogenes, such as *RAS* and *BRAF*, accounts for the high proliferation rate and increased aggressiveness of the more aggressive forms of thyroid cancer [73].

### 2.6. Other Molecular Alterations in Thyroid Cancer

**EIF1AX:** Mutations in the *eukaryotic translation initiation factor 1A* (*EIF1AX*) cause defects in the formation of the 43S pre-initiation complex for protein translation (Figure 3). Alterations of this gene are clustered in exons 2, 5 and 6, and the most common is the A113 splice mutation at the intron 5/exon 6 splice site, followed by a cluster of mutations in exon 2 [74]. *EIF1AX* mutations were detected in 1–2% of PTCs, largely occurring in a mutually exclusive manner with *BRAF* and *RAS* mutations [30], and more frequently in PDTCs (11%) and ATCs (9%) in which, on the contrary, they are strongly associated with *RAS* mutations [39].

**IDH1:** Mutations in the *isocitrate dehydrogenase 1* (*IDH1*) gene are highly prevalent in thyroid carcinoma (16%) in particular in FTCs (5%) and ATCs (11%) [75,76] (Figure 3). Even if the most recurrent IDH1 mutation involves arginine at codon 132, no R132 amino acid change has been reported in thyroid tumors. All IDH1 substitutions found in thyroid samples concern five hotspot mutations in exon 4: G70D, G123R, I130M, H133Q and A134D. For some of these mutants, a reduced enzymatic activity has been demonstrated, suggesting a potential tumorigenic role of the IDH1 system in thyroid cancer [76].

***PPARγ:****Peroxisome proliferator activated receptor gamma* (*PPAR-γ*) is a nuclear transcription factor that enhances apoptosis by activating caspases, up-regulating Bax and down-regulating bcl-2, survivin and c-myc [77,78]. Besides *RAS* mutations, rearrangements of the *PPARγ* gene are the most frequent alterations found in FTCs (20–50%) and may represent an initiating event in the transformation of follicular-derived cells [79]. *PPARγ* fusions may involve *PAX8* and *CREB3L2*, but the *PAX8-PPARγ* rearrangement is the most frequent [35,80]. The PAX8-PPAR fusion protein (PPFP) acts as a dominant negative inhibitor of wild type PPARγ, thereby constitutively activating the transcription of a subset of PPARγ and PAX8 responsive genes [80] (Figure 3).

**TERT:** Activating mutations in the promoter of the *telomerase reverse transcriptase* (*TERT*) are mostly a late event in thyroid tumorigenesis [39,81]. They are more common in PDTCs (40%) and ATCs (70%), even if they can also be found in PTCs (10%) and FTCs (20%) [81], where they are associated with a poor prognosis [82]. Two mutually exclusive *TERT* promoter mutations are recurrent in thyroid cancer, one at position -124 (c228t) and one at position -146 (c250t) upstream of the *TERT* translation start site. Both mutations generate a consensus-binding site in the *TERT* promoter for E-twenty-six (ETS) transcription factors, which increase TERT transcriptional activities [81] (Figure 3). Finally, *TERT* mutations may co-occur with *BRAF* and *RAS* mutations in PDTCs and ATCs [82,83].

Additional alterations occurring in advanced follicular-derived thyroid cancer concern: (i) Members of the DNA Mismatch Repair pathway (**MSH2**, **MSH6**, and **MLH1**), mutated in 2% of PDTCs and 12% of ATCs that induce a “hypermutator phenotype”; (ii) histone methyl-transferases (**HMTs**), altered in 7% of PDTCs and 24% of ATCs; (iii) genes encoding for members of the **SWI-SNF** chromatin remodeling complex, mutated in 6% of PDTCs and 36% of ATCs [39]. Finally, specific histone deacetylase (**HDAC**) subtypes are associated with different thyroid cancer characteristics and behaviors [84] (Figure 3).

## 3. Targeted Therapies in Thyroid Cancer

Due to an improved understanding of thyroid cancer biology, in recent years, a plethora of targeted molecules have been tested while other compounds are currently under investigation. Herein, we thoroughly review the current landscape of targeted therapies for thyroid cancer. Ongoing trials awaiting preliminary results are also reported.

### 3.1. Tyrosine Kinase Inhibitors

#### 3.1.1. Multi-Target Agents

**Figure 4:**
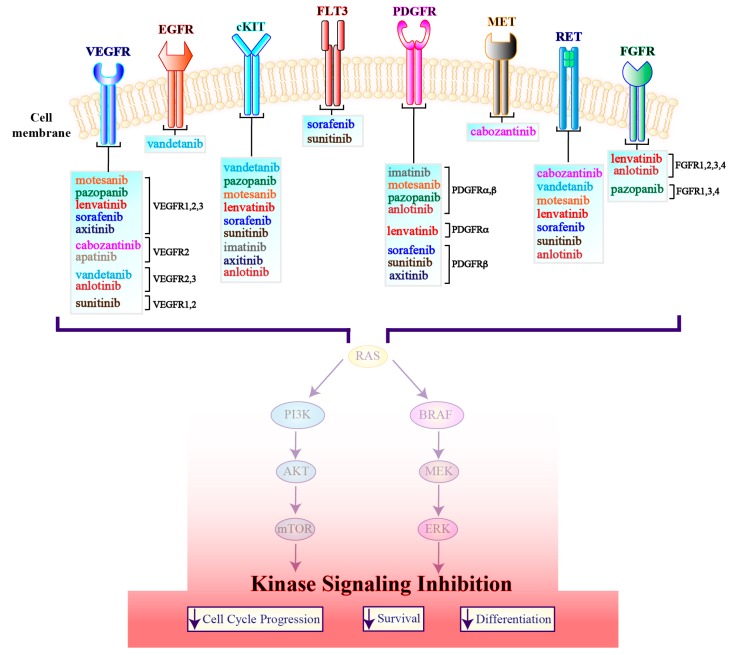
A working model describing vertical inhibition of cell cycle progression, survival and differentiation by agents targeting tyrosine kinase receptors. Multi-targeted kinase inhibitors included in each box have been investigated in thyroid cancer and are cataloged according to their receptor kinase specificity and selectivity. VEGFR, vascular endothelial growth factor receptor; EGFR, epidermal growth factor receptor; c-KIT, Mast/Stem Cell Growth Factor Receptor Kit; FLT3, FMS-like tyrosine kinase 3; PDGFR, platelet-derived growth factor receptor; MET, proto-oncogene, receptor tyrosine kinase; RET, rearranged during transfection; FGFR, fibroblast growth factor receptor; PI3K, phosphoinositide 3-kinase; AKT, V-Akt Murine Thymoma Viral Oncogene Homolog; mTOR, Mammalian Target of Rapamycin; BRAF, B-Raf proto-oncogene; MEK, Mitogen-Activated Protein Kinase; ERK, Extracellular Signal-Regulated Kinase.

**Figure 5:**
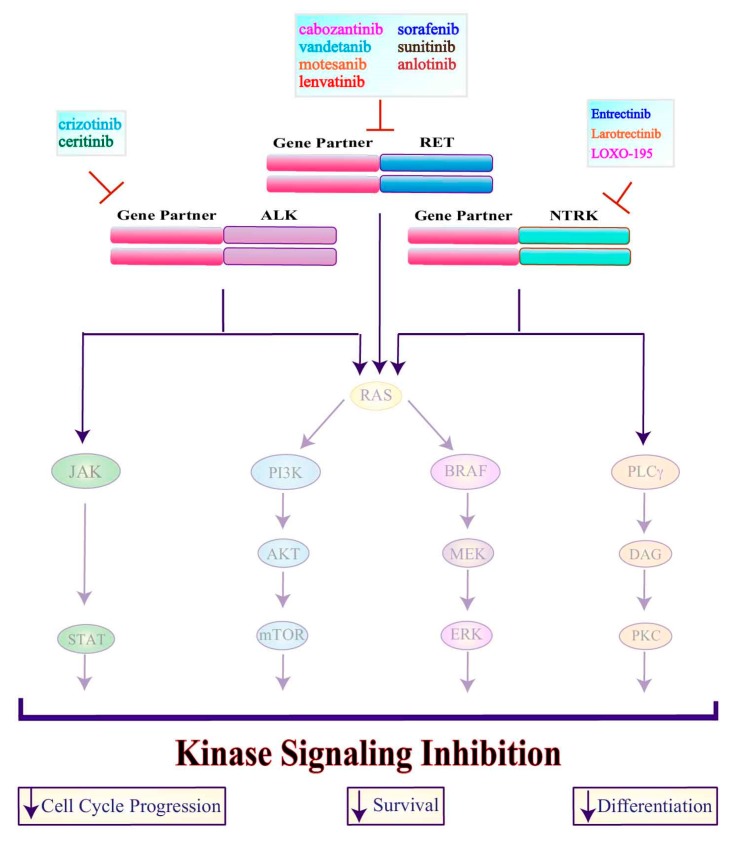
Mechanism of action of ALK, NTRK and RET fusion proteins inhibitors. Compounds targeting the ALK, NTRK and RET fusion proteins are listed in boxes. These drugs inhibit multiple cellular processes blocking cell cycle progression, survival and differentiation. ALK, anaplastic lymphoma kinase; NTRK, neurotropic tropomyosin receptor kinase; RET, rearranged during transfection; RAS, Rat Sarcoma; JAK, Janus kinase; STAT, signal transducers and activators of transcription; PI3K, phosphoinositide 3-kinase; AKT, V-Akt Murine Thymoma Viral Oncogene Homolog; mTOR, Mammalian Target of Rapamycin; BRAF, B-Raf proto-oncogene; MEK, Mitogen-Activated Protein Kinase; ERK, Extracellular Signal-Regulated Kinase; PLCγ, phospholipase C-γ; DAG, Diacylglycerol; PKC, protein kinase C.

Several molecules inhibiting tyrosine kinases involved in cell proliferation, survival and angiogenesis have shown clinical efficacy in both advanced RAIR DTCs and MTCs, while promising results are also beginning to emerge in ATCs (Figure 4 and Figure 5). To date, four drugs have received FDA approval, two for advanced RAIR DTCs (sorafenib and lenvatinib) and two for metastatic MTCs (vandetanib and cabozantinib). However, numerous trials have investigated additional tyrosine kinase inhibitors (TKIs), whereas many others are currently ongoing (Table 1). 

***Anlotinib:*** A multi-target TKI, displaying an affinity for VEGFR 2-3, FGFR 1-4, PDGFR α/β, c-KIT and RET [85]. After a phase I trial defined an MTD of 12 mg daily in a 2/1 schedule, a phase IIA study tested the drug on 58 chinese patients with advanced MTC. Partial responses (PR) occurred in 57% of patients, whereas median PFS was not reached at the time of data cut-off [85,86]. Results from a phase IIB randomized trial on 91 metastatic MTC patients were recently reported: mPFS was 20.7 months in the experimental arm and 11 months in the placebo arm (HR 0.53; *p <* 0.03), with a considerable benefit in terms of ORR (48% with anlotinib vs. 3.5% with placebo, *p* < 0.0001). Overall survival data are still immature. Adverse events were consistent throughout the trials, with hand-foot syndrome, hypertension and hyper-triglyceridemia representing the more frequent toxicities [87].

***Axitinib:*** A VEGFR1-2-3 inhibitor [88] that also targets PDGFR-β and c-KIT. Its activity in thyroid tumors of any histology has been explored in two phase-II trials, comprehensively, including 112 patients. Results were consistent in the two studies, with an ORR of 30% in the first one and of 35% in the second, and median PFS of 18.1 and 16 months, respectively [89,90].

***Cabozantinib:*** A selective inhibitor of MET, VEGFR-2 and RET [91]. In a phase I trial cabozantinib showed encouraging results in a cohort of 37 heavily pre-treated MTC patients [92]. These promising findings were further confirmed in the phase III EXAM study that randomized 330 patients with hereditary or sporadic advanced MTC to receive cabozantinib 140 mg daily or placebo, demonstrating a significant PFS benefit for subjects in the experimental arm (11.2 months vs. 4 months, HR 0.28, CI 0.19–0.40, *p* < 0.001). However, OS did not differ significantly between the two arms. The drug seems more effective in patients carrying a *RET* mutation [93,94]. In 2012 FDA approved cabozantinib for the treatment of advanced MTC [95]. More recently, the potential role of cabozantinib as salvage treatment in RAIR DTCs progressing on a VEGFR inhibitor has been explored in a phase II trial. Twenty-five enrolled patients presented a 40% ORR and a 92% disease control rate (DCR), with 12.7 months of median PFS and 34.47 months of median OS [96].

***Imatinib:*** A multiple kinases inhibitor that targets ABL, c-KIT and PDGFR. This drug was tested both in advanced or relapsed ATCs and MTCs. A pilot trial with 11 ATC patients with proven PDGFR overexpression by IHC showed that 800 mg Imatinib/daily determined 2 PR and four assessments of stable disease [97]. However, in two additional studies, a total of 24 advanced MTCs received imatinib 600 mg daily with dismal results (i.e., no OR and sporadic disease stabilization) [98,99].

***Lenvatinib:*** The drug inhibits several targets, including VEGFR 1-2-3, FGFR 1-2-3-4, PDGFR-α, RET and c-KIT [100]. Its activity in advanced RAIR DTCs has been proven in a phase II trial enrolling 58 patients, treatment naïve or pre-treated, and then confirmed in phase III SELECT trial, which randomized 261 subjects to receive 24 mg lenvatinib daily or placebo. Median PFS was 18.3 months with lenvatinib versus 3.6 months with placebo (HR 0.21; 95% CI 0.14 to 0.31; *p* < 0.0001), with a 64% OR rate, including four complete responses (CR). Median OS was not reached at the time of data cutoff. Most frequent adverse events were hypertension, diarrhea, fatigue, appetite and weight loss and nausea, with a 14% discontinuation rate [101,102]. Because of its favorable efficacy and safety profile, in 2015 lenvatinib was granted approval for the treatment of advanced RAIR DTCs and is currently the preferred therapeutic choice for this disease in the first line setting [103]. The drug also showed activity in advanced MTCs, since a phase II trial on 59 patients reported 36% OT rates (CI 24–49%) with 80% DCR (CI 67–89%) and nine months of median PFS (CI seven months-NE) [104].

***Motesanib:*** A VEGFR 1-2-3, PDGFR, RET and c-KIT inhibitor [105] that demonstrated activity in two phase II trials enrolling RAIR DTCs and MTCs, respectively. Ninety-three patients with advanced RAIR DTCs received motesanib (125 mg daily)—13 (14%) experienced a PR, whereas 33 additional patients (35%) had disease stabilization >24 weeks [106]. The second trial recruited 91 patients with advanced symptomatic or progressive MTCs: only two patients (3%) achieved a PR, while 44 (48%) had an SD >24 weeks [107].

***Pazopanib:*** A multi-kinase inhibitor targeting VEGFR 1-2-3, PDGFR-α and β, c-KIT and FGFR 1-3-4 [108]. Thirty-seven patients with RAIR DTCs, either pre-treated or TKI naïve, received the drug—at the daily dose of 800 mg—in a phase II trial. Results were encouraging, with almost 50% ORR, durable responses and a median PFS of 11.7 months [109]. Conversely, pazopanib showed negligible activity in 15 ATCs [110]. Finally, in 35 patients with progressive MTC, the drug demonstrated moderate efficacy, inducing PR in five patients (14%) and a 9.4 months median PFS [111].

***Sorafenib:*** A multi-kinase inhibitor used against VEGFR 1-2-3, RET, RAF, PDGFR-β, c-KIT and FLT3 [112]. In a single arm phase II trial, Schneider and colleagues reported 18 months of median PFS [mPFS (CI 7–29 months)] and 34.5 months median OS (CI 19–50 months) in 31 patients with advanced RAIR DTC [113]. The subsequent phase III, multicenter, randomized, double blinded, placebo-controlled DECISION trial enrolled 417 advanced RAIR DTC patients in the first line setting. It showed significantly longer PFS in the experimental arm (10.8 months) compared with the placebo arm (5.8 months) [HR 0.59; 95% CI 0.45 to 0.76; *p* < 0.0001] and a 12% ORR, but failed to demonstrate a survival benefit in the group of patients treated with sorafenib 400 mg *bis in die*, which reported considerable toxicities leading to dose reduction or treatment discontinuation in more than 60% of cases [114]. Nevertheless, sorafenib is currently approved both by the Food and Drug Administration (FDA) and European Medicines Agency (EMA) for the treatment of advanced RAIR DTC. Additionally, in a cohort of 20 pre-treated ATC patients, sorafenib induced durable PR in two patients and stable disease in five subjects, whereas it led to 1 PR and 14 disease stabilizations in 16 sporadic advanced MTCs [115,116]. However, the use of this drug in anaplastic and medullary thyroid carcinomas remains off label.

***Sunitinib:*** A compound that displays a broad spectrum of activity against VEGFR 1-2, c-KIT, RET, PDGFR-β and FLT3 [117]. Preliminary results from a phase II trial on 33 evaluable patients with advanced RAIR DTC (*n* = 26) or MTC (*n* = 7) receiving sunitinib 37.5 mg on a continuous schedule indicated that 11 patients (31%) experienced a PR and 16 patients (46%) had SD, with a median time to progression of 12 months [118]. Another phase II study of continuous sunitinib in 23 RAIR DTC patients confirmed a good activity profile in this subset of patients. In fact, ORR was 26% and clinical benefit rate (CBR) 83% [119]. Additionally, the phase II THYSU trial investigated sunitinib activity at 50 mg/daily four weeks on followed by two weeks off, in 71 previously untreated advanced thyroid carcinomas of any histology. Nine out of 39 evaluable DTC patients had a disease response (1 CR and 8 PR; ORR 22%), whereas 10 of the 24 MTC patients reported a PR (ORR 38.5%). Of the four enrolled patients with ATC, only two were evaluable and experienced disease stabilization. PFS was 13.1 months and 16.5 months in DTCs and MTCs, respectively [120].

***Vandetanib:*** A RET, VEGFR 2-3, c-KIT and EGFR inhibitor primarily tested in MTC [121,122]. Preliminary efficacy data came from a phase II trial on 30 locally advanced or metastatic hereditary MTC patients, 22 of which yielded PR or disease stabilization >24 weeks (73% DCR) with vandetanib 300 mg/daily [123]. The following phase III randomized, placebo-controlled study (ZETA trial) included 331 patients with hereditary or sporadic advanced MTC and confirmed the drug’s efficacy. Median PFS was 30.5 months in the vandetanib arm compared with 19.3 months in the placebo arm [HR 0.46, CI 0.31–0-69, *p* = 0.001], while OS results were still immature. Overall, a trend of enhanced efficacy in *RET* mutated disease emerged. Diarrhea, skin rash, nausea, hypertension and headache were the most frequent adverse reactions. Additionally, QT prolongation, potentially evolving in *torsade de pointes* and sudden death, represented an infrequent, but critical adverse event [124]. In 2011 vandetanib was the first FDA-approved drug for the treatment of symptomatic or progressive MTC in patients with unresectable, locally advanced, or metastatic disease. A special “black box” warning about QTc prolongation risk exists, hence the drug can only be prescribed by certified physicians [125]. Even though vandetanib is not indicated in advanced RAIR DTCs, it has been tested in a phase II randomized trial in this population, demonstrating a good activity profile (median PFS 11.1 months vs 5.9 months in patients receiving placebo, HR 0.63, 95% CI 0.54–0.74, *p* = 0.008) [126]. A phase III trial (VERIFY, NCT01876784) randomizing 238 subjects with RAIR DTC to receive vandetanib or placebo completed accrual, but no results are yet available.

#### 3.1.2. Single-Target Agents

**Figure 6:**
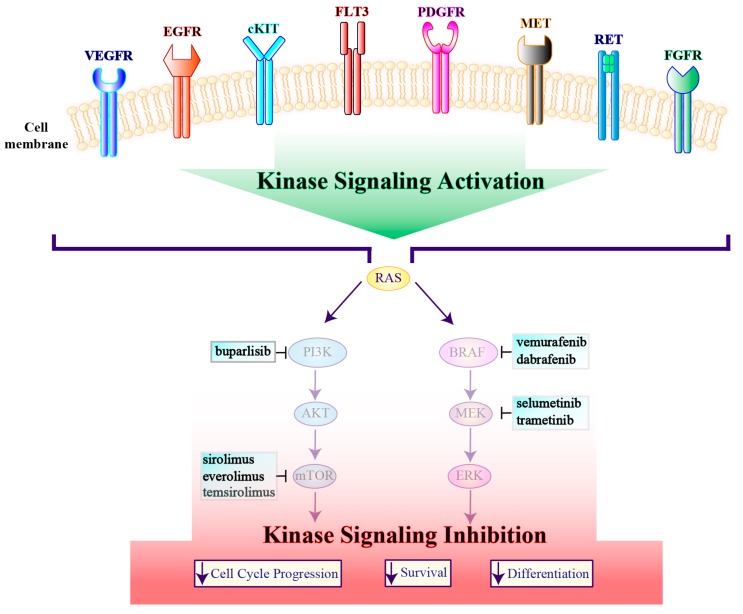
Strategies that target downstream mediators of receptor tyrosine kinases. RTK stimulation causes signaling activation that is blocked by inhibition of downstream mediators decreasing cell cycle progression, survival and differentiation. The panels report the downstream mediators inhibitors used in thyroid cancer stratified according to their molecular target specificity and selectivity. VEGFR, vascular endothelial growth factor receptor; EGFR, epidermal growth factor receptor; c-KIT, Mast/Stem Cell Growth Factor Receptor Kit; FLT3, FMS-like tyrosine kinase 3; PDGFR, platelet-derived growth factor receptor; MET, proto-oncogene, receptor tyrosine kinase; RET, rearranged during transfection; FGFR, fibroblast growth factor receptor; PI3K, phosphoinositide 3-kinase; AKT, V-Akt Murine Thymoma Viral Oncogene Homolog; mTOR, Mammalian Target of Rapamycin; BRAF, B-Raf proto-oncogene; MEK, Mitogen-Activated Protein Kinase; ERK, Extracellular Signal-Regulated Kinase.

Although multi-kinase inhibitors represent the most studied agents for the treatment of thyroid cancer, several drugs selectively blocking a single altered protein may also be effective in oncogene-addicted disease (Figure 6). Different trials have already investigated the role of single target agents, whereas many others are still ongoing (Table 2). However, in the absence of a reliable predictive biomarker, the use of single-target molecules generally leads to unsatisfactory results.

***Apatinib:*** Two small chinese trials evaluated apatinib, a selective VEGFR-2 inhibitor, in 30 patients with advanced RAIR DTC. The drug showed promising activity, both in terms of thyroglobulin levels reduction and tumor shrinkage [127,128]. Hence, further studies (phase II and III) assessing the role of apatinib for RAIR DTC treatment are currently ongoing (Table 2).

***Dabrafenib****and **Trametinib:*** Combining a BRAF inhibitor (dabrafenib) with a MEK inhibitor (trametinib) may be an effective strategy for *BRAF^V600E^* mutated ATC, as reported in a phase II trial recruiting 16 pre-treated ATC patients. The reported 69% ORR, including a CR, along with the estimated 90% rate of ongoing responses at 12 months, represent unprecedented results for this aggressive disease [129,130]. Clinical trials employing dabrafenib in combination with trametinib or lapatinib are ongoing (Table 2).

***Selumetinib:*** The drug targets MAP kinases MEK-1 and 2 and reversed iodine refractoriness in 8 of 20 patients with metastatic thyroid cancer, as assessed by ^124^I-PET. Selumetinib-treated patients subsequently received RAI achieving PR (*n* = 5) or SD (*n* = 3) after the radio-metabolic treatment. Additionally, the *NRAS* mutation seems to be a predictive biomarker of selumetinib efficacy [131]. Further studies are ongoing (Table 2).

***Vemurafenib:*** A selective BRAF inhibitor, which showed promising results in a phase II trial conducted on 51 patients with advanced RAIR PTC displaying *BRAF*^V600E^. The study population comprised two cohorts according to previous treatment with an anti-VEGFR. Ten out of 26 (38.5%) TKI naïve patients achieved a PR, whereas nine patients (35%) maintained an SD for six months or longer, determining a 73% CBR, with a median PFS of 18.2 months. In the second TKI-exposed cohort, six of the 22 evaluable patients (27%) experienced a PR, while other six had SD for at least six months (CBR 54.6%) and a median PFS of 8.9 months [132]. A further study is ongoing (Table 2).

#### 3.1.3. ALK Inhibitors

Due to its oncogenic activity, ALK represents a potential therapeutic target in many solid and hematologic cancers [133,134]. Currently, the therapeutic options for *ALK*-rearranged tumors include first- (crizotinib) or second-generation (ceritinib, alectinib and brigatinib) inhibitors or immunotherapeutic drugs directed against activated ALK (Figure 5). Ongoing trials employing ALK inhibitors are described in Table 2.

***Ceritinib:*** A second-generation inhibitor that overcomes secondary resistance, due to acquired *ALK* mutations, amplification or activation of alternative—ALK-independent—survival pathways (e.g., EGF, IGF, RAS/SRC and AKT/mTOR signaling pathways) [134,135,136]. A study by Guan at al demonstrated limited efficacy of ceritinib in ATC patients with the ALK^L1198F^ mutation in full-length ALK or the EML4-ALK fusion protein [137]. An additional trial is currently evaluating this drug in patients harboring *ALK* mutations or fusions (NCT02289144) (Table 2).

***Crizotinib:*** A second generation TKI targeting ALK, MET and ROS1. This drug has been extensively investigated in ALK-fusion-positive tumors [138]. One *ALK*-translocated ATC patient treated with crizotinib achieved a PR [139].

Jun Ho Ji and colleagues reported a dramatic response with crizotinib in an advanced MTC patient harboring an *ALK* fusion (NCT01121588) [140]. Additionally, in a phase Ib study (PROFILE 1013; NCT01121588), which enrolled 44 ALK-positive metastatic patients, one individual diagnosed with MTC experienced a PR lasting 16.1 weeks [138].

#### 3.1.4. NTRK Inhibitors

Selective inhibition of TRK signaling may be useful for patients with thyroid tumors that harbor an oncogenic *NTRK* translocation. Currently, four first and second generations TRK-inhibitors (entrectinib, larotrectinib, LOXO-195 and TPX-0005) have been developed and tested in clinical trials [141,142] (Table 2) (Figure 5).

***Entrectinib:*** A pan-TRK inhibitor with additional activity against ROS1 and ALK [143]. A phase I/Ib study in children or young adults (aged 2–22 years) with or without *NTRK*, *ROS1* or *ALK* fusions (STARTRK-NG, NCT02650401) and a phase II “basket trial” in adults with the same rearrangements (STARTRK-2, NCT02568267) enrolled patients with thyroid cancer. Results are still pending.

***Larotrectinib:*** An ATP-competitive pan-TRK inhibitor, which received agnostic approval by the FDA for patients with advanced solid tumors harboring an *NTRK* gene fusion [144]. Fifty-five patients received larotrectinib in three different trials: A phase I in adults (NCT02122913), a phase I/II in pediatric patients (SCOUT, NCT02637687) and a phase II in adolescents and adults (NAVIGATE, NCT02576431), including five patients (9%) with thyroid cancer [145]. Although data specifically concerning the thyroid population are unavailable, promising results emerged in the intention-to-treat population, whit a 75% ORR (95% CI, 61% to 85%) and 71% of patients free from progression at 12 months. Median DOR and PFS were not reached after a median follow-up of 8.3 months and 9.9 months, respectively.

***LOXO-195:*** A selective TRK inhibitor specifically developed to overcome acquired resistance that may occur in subjects receiving larotrectinib or other TRK inhibitors [145]. LOXO-195 is currently under evaluation in a phase I/II trial (NCT03215511) enrolling patients with *NTRK*-rearranged tumors—including thyroid cancer—previously treated with a TRK inhibitor.

### 3.2. PI3K/AKT/mTOR Pathway Inhibitors

Activation of the PI3K/AKT/mTOR pathway is a common feature in thyroid cancer [31,146,147]. Preclinical and clinical data suggest that targeting this pathway can be an effective strategy for the treatment of patients with advanced RAIR DTCs and MTCs [148]. To date, several trials have employed mTOR inhibitors in thyroid cancer (Table 3), whereas the phase II MATCH studies are testing PI3K (e.g., taselisib, copanlisib) and AKT-inhibitors (e.g., capivasertib) (Figure 6). 

***Buparlisib:*** A pan-class I PI3K inhibitor that failed to show a significant PFS benefit in 43 advanced, RAIR DTCs. Despite a reduction in tumor growth, the drug did not induce any objective response in the overall population, and 48.8% of patients had progressed after six months. The decrease in tumor growth may suggest an incomplete inhibition of the PI3K oncogenic pathway [149].

***Everolimus:*** A phase II trial tested this mTOR inhibitor in 38 patients with advanced RAIR DTC. The authors reported an 81% DCR and a median PFS of 47 weeks [150]. Two additional phase II studies analyzed everolimus safety and efficacy on seven subjects with MTC, 28 patients with metastatic or locally advanced DTC and seven individuals with ATC. Five patients (71.4%) showed SD, and 4 (57.1%) had an SD lasting >24 weeks [151]. In the second study, 17 patients (65%) showed SD; in 15 of these 17 patients (58%), the response lasted >24 weeks. Median PFS and OS were 9 and 18 months, respectively [152]. A further phase II trial evaluated everolimus efficacy in patients with RAIR thyroid cancer and correlated tumor mutational profiling with response. Median PFS were 12.9, 13.1 and 2.2 for DTC, MTC and ATC cohorts, respectively, and patients with mutations in the PI3K pathway appeared to benefit most from drug treatment [153].

Since activation of the somatostatin receptor (SSTR1-5) also inhibits PI3K/AKT signaling, the somatostatin analog pasireotide has been tested in combination with everolimus [154]. Pasireotide activates SSTRs, in particular, subtype 2, which is the most expressed somatostatin receptor in thyroid cancers [155]. In a phase II trial, 19 patients with advanced MTC began pasireotide, achieving a median PFS of 36 months (95% CI 19.5–52.5). Seven patients with tumor progression received everolimus in combination with pasireotide, experiencing a median PFS of nine months (95% CI 0–21.83) [156]. Another phase II trial combining pasireotide with everolimus completed accrual and results are still awaited (Table 3).

***Sirolimus:*** A retrospective study reported that this drug combined with cyclophosphamide generated PFS rates comparable to the standard of care for RAIR DTC. One-year PFS probability was 0.45 in the sirolimus plus cyclophosphamide cohort and 0.30 in the control population [157].

***Temsirolimus:*** In a phase II trial temsirolimus was combined with sorafenib for the treatment of 36 RAIR thyroid cancer patients. Radiographic response rate was the primary endpoint. A PR was observed in 22% of cases, stable disease in 58% and progressive disease in 3% of patients. Individuals with any prior systemic treatment had a response rate of 10% compared to 38% for subjects with no prior systemic treatment [158].

### 3.3. PPAR-γ Agonist—Efatutazone

Different studies suggest that PPAR-γ agonists may inhibit tumor growth through the induction of terminal cell differentiation, cell cycle arrest, apoptosis, and angiogenesis inhibition [78,159]. Efatutazone, is a PPAR-γ agonist evaluated in combination with paclitaxel in a phase I study accruing patients diagnosed with ATC. Safety, potential effectiveness, and maximally tolerated dose were the end point of this trial. Results demonstrated that the combination was safe, with no dose-limiting toxicities (DLTs) and preliminary evidence of efficacy. Thus, these findings supported the addition of efatutazone to paclitaxel in patients with advanced ATC [160]. A further clinical trial is ongoing, but not yet recruiting (Table 2).

### 3.4. Histone Deacetylase Inhibitors—Valproic Acid 

Several studies have shown that histone deacetylase (HDAC) inhibitors display promising effects for the treatment of several malignancies as they inhibit tumor proliferation, induce apoptosis, cell cycle arrest, and cancer differentiation [161,162,163].

In a phase II study valproic acid (VA) was administered to 13 patients with RAIR thyroid cancer of follicular origin. The primary endpoint of the study was to determine VA antitumor activity by evaluating measurable tumor response and/or decreased thyroglobulin levels. VA did not decrease tumor size and only generated a modest decrease in serum thyroglobulin levels. The secondary endpoint was to determine if VA could increase RAI uptake by the tumor cells, but results were disappointing [164].

## 4. Immunotherapy Landscape in Thyroid Cancer

In the last decade, the detailed understanding of the mechanism employed by cancer cells to elude the immune system have fostered a renewed interest for immune based-therapies [165]. Although thyroid carcinomas are not deemed to be “immunogenic” and display a low median tumor mutational burden (about 0.4 mutations/Mb) [166], several observations indicate a possible rationale for the use of immune checkpoint inhibitors in these tumors [167]. Indeed, immune cell infiltration, including natural killer cells, macrophages, mast cells, dendritic cells and T regulatory cells (Tregs) [168,169,170] has been reported in DTCs. Importantly, the relative amount of tumor-infiltrating lymphocytes and T-regs in primary thyroid cancer seems to correlate with prognosis [168,171]. Furthermore, the expression levels of programmed death 1 (PD-1) and programmed death ligand (PD-L1) correlate with higher risk of disease recurrence and reduced DFS [172,173]. To date, PD-L1 expression represents the most useful predictive biomarker to determine immunotherapy efficacy in thyroid tumors [168,174,175], with PD-L1 positivity ranging from 6.1 to 82.5% in PTCs and from 22.2% to 81.2% in ATCs [176].

### Immune Checkpoint Inhibitors

Several clinical trials are investigating immune checkpoint inhibitors in thyroid cancer both alone or in combination with other drugs. A phase Ib trial tested the anti PD-1 antibody pembrolizumab on 22 PD-L1-positive patients with RAIR thyroid tumors. Clinical benefit rate was 50% (95% CI 28–72%), even though seven patients experienced early progression (32%; 95% CI 14–55%). Median PFS was seven months (95% CI 2–14 months), whereas median OS was not reached at the time of data cut-off (95% CI 22 months to NR) [177]. Based on these promising results, a phase II basket trial is currently ongoing (NCT02628067). Another study is testing the combination of the anti PD-1 nivolumab and the anti CTLA-4 ipilimumab in RAIR DTCs (NCT03246958).

Immunotherapy is also being evaluated in ATC. However, while the results of a phase II trial employing pembrolizumab are awaited for October 2019 (NCT02688608), a recently presented study failed to demonstrate any efficacy for the combination of the anti-PD-L1 durvalumab with the anti CTLA-4 tremelimumab and stereotactic radiation therapy in 12 patients with ATC [178]. Additional clinical trials are ongoing to establish if immune checkpoint inhibitors may prove of clinical benefit for thyroid cancer (Table 4).

## 5. Conclusions

In the last few years, a rapid advance in the knowledge of the molecular mechanisms underlying thyroid tumorigenesis along with the identification of pivotal driver genes contributing to disease progression has led to the introduction of several biological therapies, including TKIs, monoclonal antibodies and antibody-drug conjugates [179,180,181,182,183].

In this wide landscape of potentially targetable genomic alterations, RTKs modulating angiogenesis, proliferation and differentiation have represented the most easily druggable targets. Indeed, small molecules blocking these receptors have provided significant survival benefits for both RAIR DTCs and MTCs. However, these benefits have come at the cost of meaningful clinical and financial toxicities [184,185].

While additional studies are currently investigating other RTK-directed TKIs in DTCs and MTCs, the use of these drugs in ATC has been largely unsatisfactory. On the contrary, the combination of BRAF and MEK inhibitors has generated unprecedented response rates in patients diagnosed with these aggressive thyroid carcinomas, and validation of the published preliminary results is eagerly awaited with the hope that it may provide durable benefits comparable to those reported in *BRAF*-mutant melanoma.

Finally, despite not being classified as a highly immunogenic, preliminary findings suggest a possible benefit from immune checkpoint inhibitors in thyroid cancer, although these data are still immature. As in other types of solid tumors, it remains to be established which patients will derive meaningful benefits from this therapeutic approach [186].

## Figures and Tables

**Table 1:** Clinical trials with multi-kinase inhibitors.

AGENT	COMBINATION	STUDY POPULATION	DESIGN	PATIENTS	PRIMARY END POINT	STATUS	IDENTIFIER
Cabozantinib Lenvatinib Sorafenib Vandetanib		Advanced TC	Nonrandomized, Open label, phase II	45	PFS, OS, DLTs	Recruiting	NCT03630120
Cabozantinib		RAIR DTC pretreated with anti-VEGFR	Randomized, Double blind, phase III	300	PFS, ORR	Recruiting	NCT03690388
Cabozantinib		RAIR DTC	Nonrandomized, Open label, phase II	43	SP	Active, not recruiting	NCT02041260
Cabozantinib	Ipilimumab Nivolumab	RAIR DTC pretreated with anti-VEGFR	Nonrandomized, Open label, phase II	24	ORR	Not yet recruiting	NCT03914300
Cabozantinib	Atezolizumab	LA or M+ Solid Tumors	Nonrandomized, Open label, phase Ib	1000	DLTs, ORR	Recruiting	NCT03170960
Cabozantinib		Recurrent or Refractory Solid Tumors	Nonrandomized, Open label, phase I	41	DLTs	Active, not recruiting	NCT01709435
Imatinib		RAIR PTC	Nonrandomized, Open label, phase I	18	EP	Recruiting	NCT03469011
Lenvatinib		Advanced RAIR TC	Randomized, Double blind, phase II	152	ORR, SP	Recruiting	NCT02702388
Lenvatinib		ATC	Nonrandomized, Open label, phase II	34	ORR	Terminated	NCT02657369
Lenvatinib		ATC	Nonrandomized, Open label, phase II	39 *	OS	Active, not recruiting	NCT02726503
Lenvatinib	Denosumab	Bone M+ RAIR DTC	Nonrandomized, Open label, phase II	35	EP	Not yet recruiting	NCT03732495
Lenvatinib	RAI	RAI-sensitive DTC	Nonrandomized, Open label, phase II	30	PFS	Recruiting	NCT03506048
Lenvatinib	Pembrolizumab	RAIR DTC	Nonrandomized, Open label, phase II	60	ORR	Recruiting	NCT02973997
Pazopanib		TC	Randomized, Open label, phase II	168	PFS	Recruiting	NCT01813136
Pazopanib		Advanced TC	Nonrandomized, Open label, phase II	152	ORR	Active, not recruiting	NCT00625846
Pazopanib	Paclitaxel RT	ATC	Randomized, Open label, phase II	121 *	OS, DLTs	Active, not recruiting	NCT01236547
Sorafenib		Adjuvant after RAI	Nonrandomized, Open label, phase II	32	ORR	Completed	NCT00887107
Sorafenib		Advanced.TC	Nonrandomized, Open label, phase II	61	ORR	Completed	NCT00654238
Sorafenib		Advanced.TC	Nonrandomized, Open label, phase II	25	ORR	Terminated	NCT00095693
Vandetanib		Hereditary MTC	Nonrandomized, Open label, phase I/II	17	SP	Active, not recruiting	NCT00514046
Vandetanib		Advanced MTC	Randomized, Double blind, phase III	437	PFS	Active, not recruiting	NCT00410761

Anaplastic thyroid cancer (ATC); Dose-limiting toxicities (DLTs); Differentiated thyroid cancer (DTC); Efficacy profile (EP); Locally advanced (LA); Medullary thyroid cancer (MTC); Metastatic (M+); Objective response rate (ORR); Overall survival (OS); Pharmacodynamic (PD); Pharmakinetics (PK); Progression free survival (PFS); Papillary Thyroid Cancer (PTC); Radioactive iodine (RAI); Radioactive iodine resistance (RAIR); Radio therapy (RT); Safety profile (SP); Thyroid cancer (TC). * number of estimated patients.

**Table 2:** Clinical trials with single target agents.

TARGET	AGENT	COMBINATION	STUDY POPULATION	DESIGN	PATIENTS	PRIMARY END POINT	STATUS	IDENTIFIER
ALK	Alectinib		RET-rearranged NSCLC or RET-mut TC	Non-Randomized, Open Label, Phase I/II	78*	MTD, ORR	Active, not recruiting	NCT03131206
	Ceritinib		M+ or LA ATC	Single Group Assignment, Open Label, Phase II	100*	Development of progression	Recruiting	NCT02289144
BRAF	Dabrafenib	Trametinib	Recurrent TC	Randomized, Open label, phase II	53	ORR	Active, not recruiting	NCT01723202
	Dabrafenib	Trametinib RAI	M+ RAIR with RAS or BRAF mutation	Nonrandomized, Open label, phase II	87	ORR	Recruiting	NCT03244956
	Dabrafenib	Lapatinib	TC with BRAF mutation	Nonrandomized, Open label, phase I	18	DLTs	Active, not recruiting	NCT01947023
	Vemurafenib		Neoadjuvant-Advanced TC	Nonrandomized, Open label, phase II	24	EP	Active, not recruiting	NCT01709292
MEK	Selumetinib	Olaparib	Solid tumors with Ras pathway alterations, and ovarian tumors with PARP resistance	Nonrandomized, Open label, phase I	90	DLTs	Recruiting	NCT03162627
	Selumetinib	^131^I	Recurrent or M+ TC	Randomized, Double blind, phase II	60	ORR	Recruiting	NCT02393690
	Trametinib	Paclitaxel	Advanced ATC	Nonrandomized, Open label, phase I	12	PFS	Recruiting	NCT03085056
	Trametinib	Pazopanib	Advanced Solid Tumors (DTC, STS and Chol)	Nonrandomized, Open label, phase I	89	DLTs, SP	Completed	NCT01438554
	Trametinib	RAI	RAS mutant or RAS/RAF wild-type, RAIR and/or M+ TC	Nonrandomized, Open label, phase II	35	PFS, ORR	Recruiting	NCT02152995
NTRK	Entrectinib		LA or M+ Solid Tumors harboring NTRK1/2/3, ROS1, or ALK Rearrangements	Non-Randomized, Open Label, Phase II	300*	ORR	Recruiting	NCT02568267
	Entrectinib		Solid tumors with or without TRK, ROS1 or ALK Fusions	Non-Randomized, Open label, Phase I	65*	MTD, RP2D, ORR	Recruiting	NCT02650401
	Larotrectinib		Solid Tumors Harboring NTRK Fusion	Non-Randomized, Open Label, Phase II	320*	ORR	Recruiting	NCT02576431
	LOXO-195		Patients with previously treated NTRK Fusion cancers	Single Group Assignment, Open label, Phase I/II	93*	MTD, recommended dose, PR, CR	Recruiting	NCT03215511
PPAR-γ	Efatutazone	Paclitaxel	Advanced ATC	Nonrandomized, Open label, phase II	19	ORR	Active, not Recruiting	NCT02152137
VEGFR-2	Apatinib	RT	Inoperable or RAIR TC	Nonrandomized, Open label, phase II	20	PFS	Recruiting	NCT03300765
	Apatinib		RAIR DTC	Randomized, Double blind, phase III	118	PFS	Recruiting	NCT03048877
	Apatinib		Locally Advanced/M+ DTC	Nonrandomized, Open label, phase II	20	EP	Recruiting	NCT03167385
	Apatinib		Local Progressive/M+ RAIR	Nonrandomized, Open label, phase II	40	ORR	Recruiting	NCT03199677

Anaplastic thyroid cancer (ATC); Cholangiocarcinoma (Chol); Complete response (CR); Dose-limiting toxicities (DLTs); Differentiated thyroid cancer (DTC); Efficacy profile (EP); Locally advanced (LA); Maximum Tolerated Dose (MTD); Medullary thyroid cancer (MTC); Metastatic (M+); Non small cell lung cancer (NSCLC); Objective response rate (ORR); Overall survival (OS); Pharmacodynamic (PD); Pharmacokinetics (PK); Progression-free survival (PFS); Partial response (PR); Radioactive iodine (RAI); Radioactive iodine resistance (RAIR); Radiation therapy (RT); Recommended phase 2 dose (RP2D); Safety profile (SP); Soft tissue sarcoma (STS); Thyroid cancer (TC). * number of estimated patients.

**Table 3:** Clinical trials with mTOR inhibitors.

AGENT	COMBINATION	STUDY POPULATION	DESIGN	PATIENTS	PRIMARY END POINT	STATUS	IDENTIFIER
Everolimus		LA or M+ TC	Nonrandomized, Open label, phase II	40	ORR	Completed	NCT01164176
Everolimus		RAIR TC	Nonrandomized, Open label, phase II	33	PFS	Active, not Recruiting	NCT00936858
Everolimus	Lenvatinib	M+ DTC progressed on Lenvatinib alone	Nonrandomized, Open label, phase II	40	PFS	Recruiting	NCT03139747
Everolimus	Neratinib	Advanced Cancer with EGFR/HER2 Mut/Ampl, HER3/4 Mut	Nonrandomized, Open label, phase I	120	DLTs	Recruiting	NCT03065387
Everolimus	Pasireotide	RAIR DTC and MTC	Randomized, Open label, phase II	42	ORR	Completed	NCT01270321
Everolimus	Sorefenib	M+ DTC progressed on Sorafenib alone	Nonrandomized, Open label, phase II	40	ORR, PFS	Active, not Recruiting	NCT01263951
Everolimus	Sorefenib	Advanced TC never treated with m-TOR inhibitor or Sorafenib	Nonrandomized, Open label, phase II	41	ORR	Active, not Recruiting	NCT01141309
Everolimus	Sorefenib	Advanced RAIR Hurthle Cell TC	Randomized, Open label, phase II	34l	PFS	Recruiting	NCT02143726
Everolimus	Vatalinib	Advanced Solid Tumors	Nonrandomized, Open label, phase I	96	DLTs, SP	Completed	NCT00655655
Sirolimus	Ciclofosfamide	M+ or RAIR DTC	Nonrandomized, Open label, phase II	19	ORR	Recruiting	NCT03099356
Sirolimus	Grapefruit juice	Advanced Malignancies	Nonrandomized, Open label, phase Ib	41	PK	Completed	NCT00375245
Temsirolimus	Bevacizumab Valproic Acid	Advanced or M+ Malignancy or Other Benign Disease	Nonrandomized, Open label, phase I	216	DLTs	Recruiting	NCT01552434
Temsirolimus	Vinorelbine	Unresectable or M+ Solid Tumors	Nonrandomized, Open label, phase I	19	DLTs, ORR	Completed	NCT01155258

Dose-limiting toxicities (DLTs); Differentiated thyroid cancer (DTC); Locally advanced (LA); Medullary thyroid cancer (MTC); Metastatic (M+); Objective response rate (ORR); Pharmacokinetics (PK); Progression-free survival (PFS); Radioactive iodine resistance (RAIR); Safety profile (SP); Thyroid cancer (TC).

**Table 4:** Clinical trials with immune checkpoint inhibitors.

TARGET	AGENT	COMBINATION	STUDY POPULATION	DESIGN	PATIENTS	PRIMARY ENDPOINT	STATUS	IDENTIFIER
PD-1	Pembrolizumab		M+ or LA ATC	Single Group Assignment Open label, phase II	20	RR	Recruiting	NCT02688608
	Pembrolizumab		Recurrent or M+ MTC	Nonrandomized Parallel Assignment Open label, phase II	32	DLTs	Recruiting	NCT03072160
	Pembrolizumab		Patients with rare cancer types	Single Group Assignment, Open label, phase II	350	ORR	Recruiting	NCT03012620
	Pembrolizumab		Advanced Solid Tumors	Single Group Assignment, Open label, phase II	1350	ORR	Recruiting	NCT02628067
	Pembrolizumab	Docetaxel	Poorly Chemo-responsive Thyroid and Salivary Gland Tumors	Nonrandomized, Parallel Assignment Open label, phase I	46	RR	Recruiting	NCT03360890
	Pembrolizumab	Docetaxel Doxorubicin	ATC	Nonrandomized, Open label, phase II	3*	OSR	Active, not Recruiting	NCT03211117
	Pembrolizumab	Lenvatinib	RAIR DTC	Single Group Assignment, Open label, phase II	60	CRR	Recruiting	NCT02973997
PD-1 and CTLA-4	Nivolumab Ipilimumab		RAIR DTC, ATC, MTC	Randomized, Parallel Assignment Open label, phase II	54	RRR	Recruiting	NCT03246958
PD-L1	Atezolizumab	Bevacizumab Cobimetinib Paclitaxel Vemurafenib	ATC, PDTC	Nonrandomized, Parallel Assignment Open label, phase II	50	OS	Recruiting	NCT03181100
	Atezolizumab	Cabozantinib	M+ ATC	Nonrandomized Sequential Assignment Open label, phase I-II	1000	DLTs	Recruiting	NCT03170960
	Durvalumab		TC	Single Group Assignment Open label, phase I	11	DLTs	Recruiting	NCT03215095
PD-L1 and CTLA-4	Durvalumab+ Tremelimumab		M+ ATC	Single Group Assignment Open label, phase	13	OS	Active, not Recruiting	NCT03122496

Anaplastic thyroid cancer (ATC); Complete remission rate (CRR); Dose-limiting toxicities (DLTs); Differentiated thyroid cancer (DTC); Locally advanced (LA); Medullary thyroid cancer (MTC); Metastatic (M+); Objective response rate (ORR); Overall survival (OS); Overall survival rate (OSR); Poorly differentiated thyroid cancer (PDTC); Radioactive iodine resistance (RAIR); Radiographic Response Rate (RRR); Response rate (RR). * number of actual patients.
